# Successful Pregnancy in a Woman of Advanced Maternal Age at Sixteen Months Post-Roux-en-Y Gastric Bypass Revision

**DOI:** 10.7759/cureus.34966

**Published:** 2023-02-14

**Authors:** Srijesa Khasnabish, Dondre Irving, Seyed Mohammad Nahidi, Christopher Engler, Leaque Ahmed

**Affiliations:** 1 Internal Medicine, New York Institute of Technology College of Osteopathic Medicine, New York, USA; 2 Surgery, Wyckoff Heights Medical Center, Brooklyn, USA; 3 Internal Medicine, Wyckoff Heights Medical Center, Brooklyn, USA

**Keywords:** obesity, laparoscopic roux-en-y gastric bypass, pregnancy, advanced maternal age, bariatric surgery complications

## Abstract

Obesity, defined as body mass index (BMI) > 30 kg/m^2^, complicates maternal and neonatal outcomes. Bariatric surgery (BS) is an option for weight reduction in several populations, including reproductive-aged women. However, there is a lack of consensus regarding the ideal time interval between BS and pregnancy.

We report the case of a 43-year-old Hispanic female who underwent an initial Roux-en-Y gastric bypass (RYGB) in 2011, followed by a revision eight years later in 2019. The revision entailed the reduction of the gastric pouch size and the excision of the remnant stomach. It occurred sixteen months before the conception of her second pregnancy.

Despite advanced maternal age and nutritional challenges following BS, this patient delivered a healthy male neonate and maintained a net weight loss compared to her preoperative weight. Factors leading to this positive outcome included the patient's adherence to dietary recommendations following the procedure and using weight loss adjuncts (phentermine and topiramate) to promote post-procedure weight loss.

Sixteen months between RYGB revision and conception can lead to positive pregnancy outcomes, even in women of advanced maternal age and multiple prior BS. Further studies are required to understand better the optimal interval to reduce maternal and neonatal complications following RYGB specifically and the use of medications as weight loss adjuncts.

## Introduction

Approximately 62.5% of adults in the Americas are overweight or obese [1]. In the USA, candidates for bariatric surgery (BS) must have a body mass index (BMI) ≥35 kg/m^2^ (regardless of presence, absence, or severity of co-morbidities) or a BMI ≥30 kg/m^2^ with type 2 diabetes, or a BMI of 30-34.9 kg/m^2^ and unable to achieve sustainable weight loss or co-morbidity improvement via nonsurgical methods [2]. Options for BS include procedures that restrict stomach size (laparoscopic sleeve gastrectomy [LSG]), limit nutrient absorption (biliopancreatic diversion), or both (Roux-en-Y gastric bypass [RYGB]). Biliopancreatic diversion with duodenal switch is a procedure that involves both food restriction and decreased absorption, similar to RYGB. Compared to laparoscopic RYGB, LSG is a simpler procedure as it does not require a gastrointestinal anastomosis or intestinal bypass [3]. Between 1998 - 2005, 83% of BS procedures in the United States were performed on women of reproductive age (18-45 years old) [4].

Obesity is detrimental to fertility because it interferes with normal ovarian and endometrial physiology [5]. It can also increase the risk of gestational diabetes mellitus, preeclampsia, cesarean delivery, and infectious morbidity [6]. While one can understand why women with obesity may choose to undergo BS before conception, the procedure alone is not a treatment for infertility [5]. BS can lead to severe nutritional deficiencies if patients do not adhere to the appropriate diet postoperatively [6].

We report the case of a pregnancy in a woman of advanced maternal age that resulted in positive outcomes despite an interval of only sixteen months between RYGB revision and conception.

A draft of this article was previously posted to the Research Square preprint platform on September 28, 2022. 

## Case presentation

We present the case of a 43-year-old Hispanic female who was G2P2A0 and eight years status post an RYGB procedure. Sixteen months before conception, she underwent an RYGB revision in 2019 to treat morbid obesity due to weight recurrence. The revision entailed the reduction of the gastric pouch to approximately four centimeters in length and the narrowing of the pouch through the angle of His. No alterations were made to the biliopancreatic and Roux limbs. Her past medical history included treatment-resistant type 2 diabetes mellitus and microcytic anemia. Per chart review, the patient's weight before her initial RYGB in 2011 was 136.07 kg. At the time of the RYGB revision, her weight was 102.06 kg. Twelve months following the revision, she achieved her nadir weight of 87.99 kg. See table 1 for weight loss trends before and after the RYGB revision. Her hemoglobin A1c levels decreased from 5.8% to 5.6% within three months postoperatively.

**Table 1:** Trends in Weight Loss Pre and Post-Roux-en-Y Gastric Bypass Revision Trends in weight loss pre and post-Roux-En-Y gastric bypass (RYGB) revision. The RYGB revision occurred in March 2019.

Date	Months Before or After Roux-en-Y Gastric Bypass Revision	Weight (kg)	BMI	Use of Weight Loss Adjuncts
January 2019	-2	107.96	38.4	None
March 2019	0	102.06	36.3	None
June 2019	3	97.52	34.7	15 mg phentermine
October 2019	6	95.25	33.9	30 mg phentermine
March 2020 (telehealth appointment)	12	87.99	31.3	37.5 mg phentermine
June 2020 (telehealth appointment)	15	87.54	31.1	37.5 mg phentermine and started 25 mg topiramate twice a day.
June 2021 (2 months after childbirth)	27	103.87	37.0	37.5 mg phentermine and 25 mg topiramate twice a day.
March 2022	36	95.25	33.9	37.5 mg phentermine and 25 mg topiramate twice a day.
January 2023	46	91.62	32.6	37.5 mg phentermine and 25 mg topiramate twice a day.

Due to inadequate weight loss, the patient prescribed 15 mg of phentermine at three months postoperatively in June 2019. Given that the patient tolerated this dose without any side effects, it was increased to 30 mg six months postoperatively and 37.5 mg seven months postoperatively. Fifteen months postoperatively, the patient was started on 25 mg of topiramate twice daily to facilitate further weight loss. The patient discontinued phentermine and topiramate upon confirmation of pregnancy in July 2020. Follow-up appointments were limited by the onset of the Coronavirus disease-2019 (COVID-19) pandemic in March 2020.

Although her first child was delivered via cesarean section, the patient experienced an uneventful planned pregnancy and gave birth at 40 weeks to a healthy male via the vaginal route 25 months after the RYGB revision. The patient's obstetrician provided dietary guidelines and instructed her to gain no more than 13.61 kg during her pregnancy, and the patient reported a 15.88 kg weight gain during this time. Two months postpartum (27 months postoperatively), she weighed 103.42 kg, approximately 4.54 kg below her preoperative weight.

## Discussion

Obesity is defined as BMI >30 and further subdivided into Class 1, BMI 30 to <35; Class 2, BMI 35 to < 40; and Class 3, BMI ≥ 40 [7]. A diagnosis of obesity during pregnancy is based on the pre-pregnancy BMI [8]. Given the growing prevalence of obesity in females ages 20-39, weight reduction has become an increasingly important aspect of preconception counseling [9]. Excess adipose tissue evolves into an active endocrine organ with harmful systemic effects, including insulin resistance and defective placental development [10]. Complications associated with maternal obesity during pregnancy include gestational hypertension, preeclampsia, gestational diabetes, preterm birth, and large infants for their gestational age [11]. The Barker hypothesis postulates that maternal obesity increases the propensity for adult cardiovascular disease among infants due to changes in metabolic programming in utero [12]. Thus, BS may be a suitable treatment for pre-pregnancy obesity in women who meet the established criteria.

While the American Society for Metabolic and Bariatric Surgery recommends 12-18 months after surgery and before pregnancy, there is no consensus regarding this time interval in the literature [13, 14]. Furthermore, this recommendation is not specific to the type of BS performed, the involvement of any revisions to the index surgery, or the consideration of variables about the mother. Rapid weight loss can lead to higher fertility rates by improving menstrual regularity and relieving the symptoms of the polycystic ovarian syndrome [15]. However, the dramatic weight loss following BS can hinder follicle development [16]. Studies revealed that pregnancy <12 months following RYGB was associated with a higher incidence of urinary tract infection, inadequate birth weight, and dumping syndrome compared to pregnancies initiated 12-24 months after the procedure [17]. More research will be required to understand the full impact of the length of this interval on pregnancy outcomes.

This case is unique because of our patient's advanced maternal age (43 years old) and history of two BS procedures. Fecundity begins to decline at age 32 and further declines after age 37 due to a decrease in egg quality and circulating hormone levels [18]. While we did not have access to her obstetrical records, our patient reported no difficulties with conception. Studies report that neonates born to mothers who had undergone RYGB surgery were likelier to have lower fetal growth rates [13]. Although our patient had undergone two BS procedures, she did not experience this complication. More research will be needed to understand the potential detrimental physiological and nutritional changes and their impact on pregnancies among women who have undergone BS.

Due to the start of the coronavirus-19 pandemic in March 2020, her appointment was a telehealth appointment, and this patient was lost to follow-up. Her first in-person appointment with the bariatric surgery clinic following the birth of her child was in June 2021.

The patient was treated with phentermine starting three months postoperatively; topiramate was added fifteen months postoperatively due to inadequate weight loss. The use of phentermine and topiramate as weight-loss adjuncts was approved by the United States Food and Drug Administration in 2012 [19]. Studies show increased efficacy of these weight loss medications when combined with laparoscopic sleeve gastrectomy versus RYGB. These drugs resulted in a 2.8% versus a 0.3% total body weight loss [20]. More data will be needed to understand the appropriate use of the adjuncts, particularly in reproductive-aged women. 

Patients who have undergone BS should be followed up every three months for two years to screen for nutritional deficiencies [16]. Deficiencies in vitamins A, B12, K, iron, folate, and calcium can harm the mother's and growing fetus's health. After the onset of the COVID-19 pandemic in March 2020, our patient was followed by telemedicine appointments for one year. The electronic health records documenting these encounters do not indicate any desire to conceive, although our patient's obstetrician was aware. While there are currently no specific guidelines, our patient presented at advanced maternal age and thus may have benefitted from counseling regarding pregnancy after BS.

## Conclusions

While the American Society for Metabolic and Bariatric Surgery recommends 12-18 months after surgery and prior to pregnancy, there is no consensus regarding this time interval in the literature. This case is valuable because it illustrates that a healthy pregnancy following RYGB revision is possible, even in a female of advanced maternal age with sixteen months between the procedure and conception. Furthermore, this patient sustained a net weight loss, specifically with phentermine and topiramate. Further studies are required to understand better the optimal interval to reduce maternal and neonatal complications following RYGB specifically and the use of medications as weight loss adjuncts.
